# Willingness to pay for a quality-adjusted life year among advanced non-small cell lung cancer patients in Viet Nam, 2018

**DOI:** 10.1097/MD.0000000000019379

**Published:** 2020-02-28

**Authors:** Thuy Van Ha, Minh Van Hoang, Mai Quynh Vu, Ngoc-Anh Thi Hoang, Long Quynh Khuong, Anh Nu Vu, Phuong Cam Pham, Chinh Van Vu, Lieu Huy Duong

**Affiliations:** a Viet Nam Department of Health Insurance, Ministry of Health; b Hanoi University of Public Health; c Bach Mai Hospital; d Viet Nam Health Economics Association, Hanoi, Viet Nam.

**Keywords:** 2018, non-small cell lung cancer, QALY, Viet Nam, willingness to pay

## Abstract

To examine the willingness to pay (WTP) for a quality-adjusted life year (QALY) gained among advanced non-small cell lung cancer (NSCLC) patients in Viet Nam and to analyze the factors affecting an individual's WTP.

A cross-sectional, contingent valuation study was conducted among 400 NSCLC patients across 6 national hospitals in Viet Nam. Self-reported information was recorded from patients regarding their socio-demographic status, EQ-5D (EuroQol-5 dimensions) utility, EQ-5D vas, and WTP for 1 QALY gained. To explore the factors related to the WTP, Gamma Generalized Linear Model and multiple logistic regression tools were applied to analyze data.

The overall mean and median of WTP/QALY among the NSCLC patients were USD $11,301 and USD $8002, respectively. Strong association was recorded between WTP/QALY amount and the patient's education, economic status, comorbidity status, and health utility.

Government and policymakers should consider providing financial supports to disadvantaged groups to improve their access to life saving cancer treatment.

## 1 Introduction

Lung cancer is the leading cause of cancer mortality worldwide, accounting for nearly 10 million deaths in 2018.^[1]^ Non-small cell lung cancer (NSCLC) is the most common type of lung cancer, including squamous cell carcinoma, adenocarcinoma, and large cell carcinoma, making up approximately 80% to 85% of lung cancer cases worldwide.^[2]^ NSCLC has a significant financial burden to society that increases with disease progression.^[3]^

In Viet Nam, lung cancer was reported to be the second leading cause of cancer mortality for both males and females since 2012.^[4]^ More than 80% of the lung cancer cases in Viet Nam were NSCLC, with majority of case (about 89%) Viet Nam being detected at advanced stages (IIIB or IV). A study conducted in 2014 reported that the economic burden of NSCLC in Viet Nam was more than 3517 billion VND, equivalent to $150 million. Given the significant economic burden of NSCLC in Viet Nam, cost-effective strategies for Viet Nam are needed to better manage NSCLC cases.

In Viet Nam, health technology assessments such as cost-effectiveness or cost-utility analysis has recently been applied to evaluate and recommend medicines for reimbursement as part of the health insurance scheme.^[5]^ Cost-effectiveness or cost-utility analysis estimates the incremental cost-effectiveness ratio by comparing 2 health interventions. Interventions are considered “good value for money” if the incremental cost-effectiveness ratio falls below a certain cost-effectiveness threshold. This threshold has been normally based on the level of population's willingness to pay (WTP) for a quality-adjusted life year (QALY) gained. Estimating the WTP for a QALY gained threshold among NSCLC patients would provide important information for implementation of health technology assessment to prioritize health interventions against NSCLC in Viet Nam. This study will be the first to examine the WTP for a QALY gained among advanced NSCLC patients in Viet Nam and the factors affecting WTP.

## 2 Methods

### 2.1 Study design

A cross-sectional study was conducted using contingent valuation method, a survey-based economic practice, which asks individuals how much they are willing to pay for a particular goods or service.^[6–8]^

### 2.2 Study subjects, sample size, and sampling

Patients with advanced stages of NSCLC (IIIB or IV stage) aged between 18 and 70 years were selected for this study. The sample size was estimated using the WHO formula for estimating 1 population proportion:
 
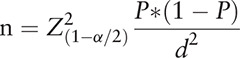


The value n defines the minimum sample size required, *P* is the anticipated proportion of NSCLC patients who were willing to pay for a QALY gained equal or above 1 GDP (gross domestic product) = 50% (proportion estimated for the largest sample), *d* is an absolute precision (.05) and *Z*_1−α/2_ = 1.96 (α = 5%). The minimum sample size was calculated to be 384. To account for non-response rate, a sample of 400 NSCLC patients were recruited for this study.

The study was conducted in the oncology departments of 6 referral hospitals in Viet Nam, which had the appropriate medical equipment for the treatment of cancer. These sites included: Bach Mai Hospital, Hanoi Oncology Hospital, Viet Nam National Cancer Hospital (in the North), Da Nang Hospital (in the Center), Cho Ray Hospital, and Ho Chi Minh City Medicine and Pharmacy University Hospital (in the South). From September to December 2018, 400 NSCLC patients, who could communicate well, were conveniently selected from these study hospitals. NSCLC patients who were unaware of their own health problem were excluded from the study. All 400 questionnaires were accepted because of no missing data and logical error.

### 2.3 Data collection and study questionnaire

Physicians from the studied hospitals were briefed on the study objectives before referring the selected patients to the interviewers. The NSCLC patients were then interviewed by trained interviewers after their routine consultation.

Patients were asked about their health states (or utility) using the EuroQol-5 dimension-5 levels instrument (EQ-5D-5L) (the Vietnamese version).^[9]^ The health utilities ranged from 1 = “perfect health” to 0 = “death”. Negative values represented health states the person considers worse than death.

To measure the patient's willingness to pay, an iterative bidding technique was applied, consisting of a sequence of dichotomous choice questions (i.e., yes or no) followed by a final open-ended question. Data collectors presented individual patients with the following question “Assuming a novel treatment method would be available now, that could free you from lung cancer and allow you to recover perfectly without any side effects, but the treatment is not covered by health insurance and you would have to pay for the treatment costs, would you be willing to pay an amount of [starting bid] per year for this kind of treatment?”

**Figure 1:**
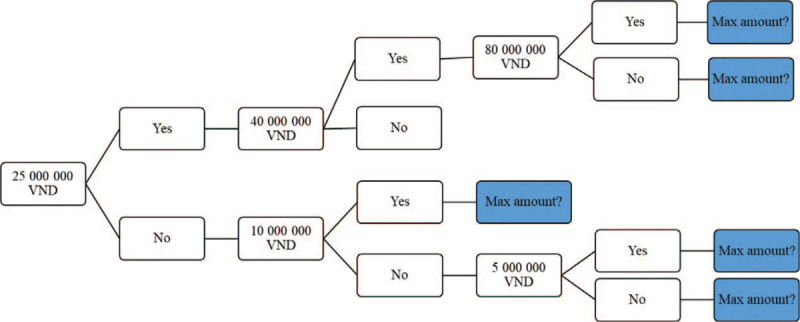
Example of iterative bidding technique with an initial bid of 25,000,000 VND.

Patients were randomly assigned bids of USD $216, $432, $1078, $1724, $2155, equating to VND 5,000,000; 10,000,000; 25,000,000; 40,000,000; 50,000,000, respectively (Table 1). These figures were benchmarked at .1; .2; .5; .7; 1 GDP per capita in Viet Nam for 2017, respectively.^[10]^ If the patient was willing to pay for the treatment at the rate of the first bid offered, then a follow-up question with a higher bid would be asked. If the respondent was unwilling to pay for the first suggested amount, then the second threshold would be reduced to a lower level. Following the double-bounded dichotomous question, all patients were presented with an open-ended question “What is the maximum price you would be willing to pay per year for the treatment?”. An example of the bidding technique is represented in Figure 1.

**Table 1:**
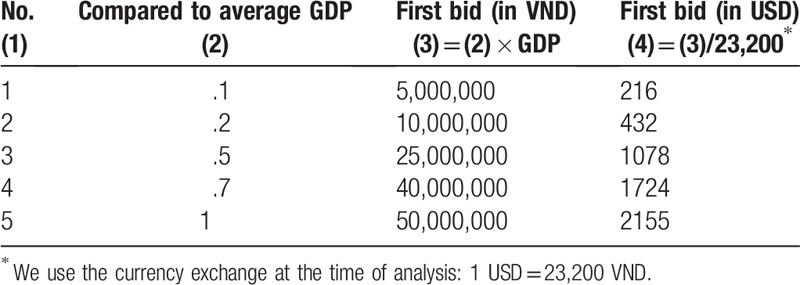
The starting bids in the iterative bidding technique.

In addition, self-reported patient's characteristics were recorded, including: sex, age, education, occupation, economic status, and health behavior such as smoking and alcohol use.

### 2.4 Data management and analysis

All study data were entered into EpiData 3.1 management software, and statistical analysis was then carried out using Stata 14. Health utility of the NSCLC patients was derived from the Viet Nam EQ-5D score set. The WTP/QALY ratio for each participant was computed using the following formula:
 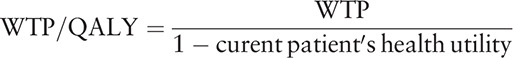


Descriptive analyses were applied to determine the background characteristics of the study participants. The generalized linear model with link (log) and gamma distribution was applied to identify individual's socio-economic traits that would influence the amount of WTP (as the data on WTP max amount were right skewed). A logistic regression model was performed, with a significance level of .05, to estimate the probability of willingness to pay for a QALY gained at the bid of equal or greater than 1 per capita GDP of Viet Nam in 2017.

### 2.5 Ethical considerations

Ethical approval was obtained from the Institutional Review Board of the Hanoi University of Public Health. Informed consent forms were obtained from all subjects before participating in the study.

## 3 Results

### 3.1 General characteristics of the study respondents

The general characteristics of the study respondents are summarized in Table 2. The study sample consisted of more men (56.3%) than women (43.8%), majority (62.3%) of the participants were over 50 years old. Most respondents (90.5%) completed secondary school or higher, with 9.5% having had an education level lower than primary school. The proportion of people who worked in formal and informal economic sectors were quite similar (49.3% and 48.8%, respectively). There were slightly more patients from rural areas (53.5%) as compared to those from urban locations (46.5%). Almost all of respondents identified themselves as the Kinh (majority group). Most of them were married (90.8%) and had no religion (87.5%). Approximately 8.3% of the patients self-identified as poor (classified by the local government). All study respondents had health insurance.

**Table 2:**
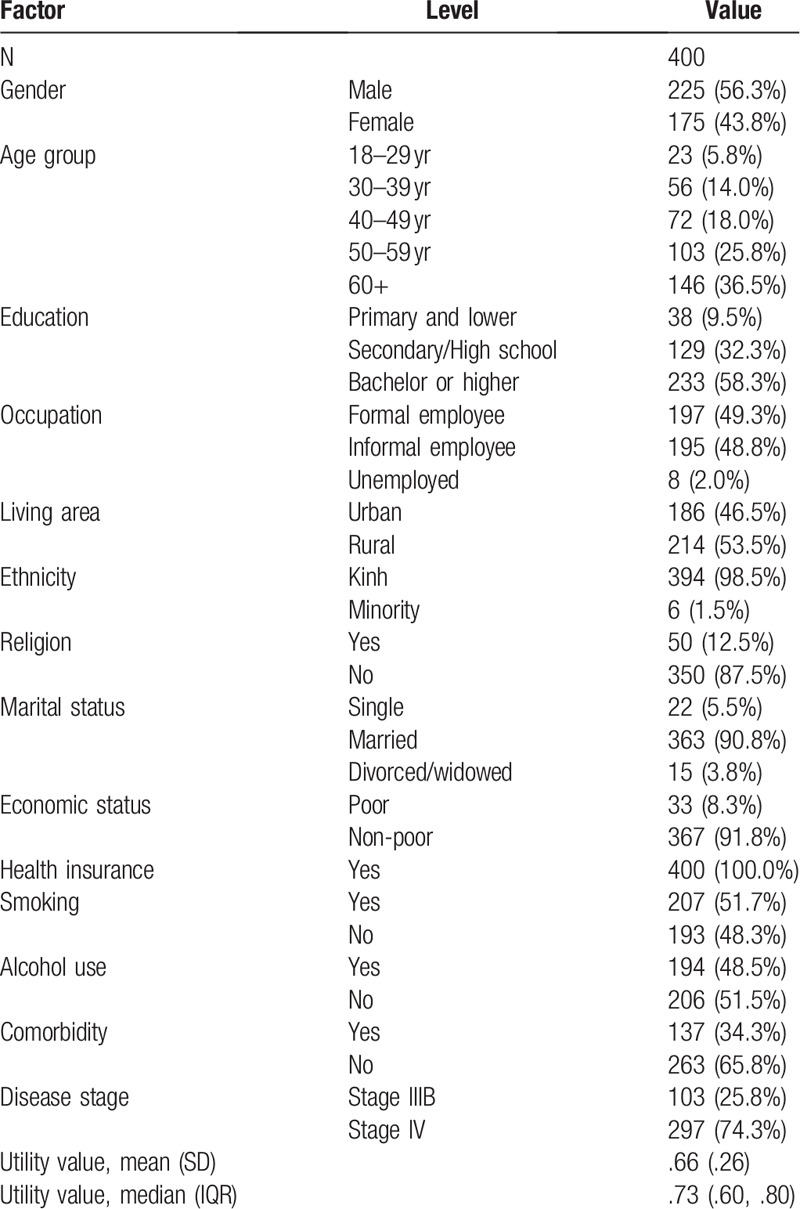
Characteristics of respondents.

The prevalence of smoking and alcohol drinking among the study respondents were 51.7% and 48.5%, respectively. The percentage of patients with disease stage IIIB and IV were 25.8% and 74.2%, respectively. About one-third of participants had other comorbidities. The mean and median of EQ-5D health utility were .66 and .73, respectively.

### 3.2 Willingness to pay for a QALY gained (WTP/QALY)

The overall mean and median of WTP/QALY among NSCLC patients were USD $11,301 and USD $8002, respectively (standard deviation of USD $11,175; with a range of USD $0 to USD $48,013). The WTP/QALY amount was identified to be higher among men, older patients, those with higher education, those who worked as formal employees, urban dwellers, Kinh people, non-poor people, non-smoking patients, non-drinking patients, patients without comorbidity, those with disease state IIIB and those with higher health utility (Table 3).

**Table 3:**
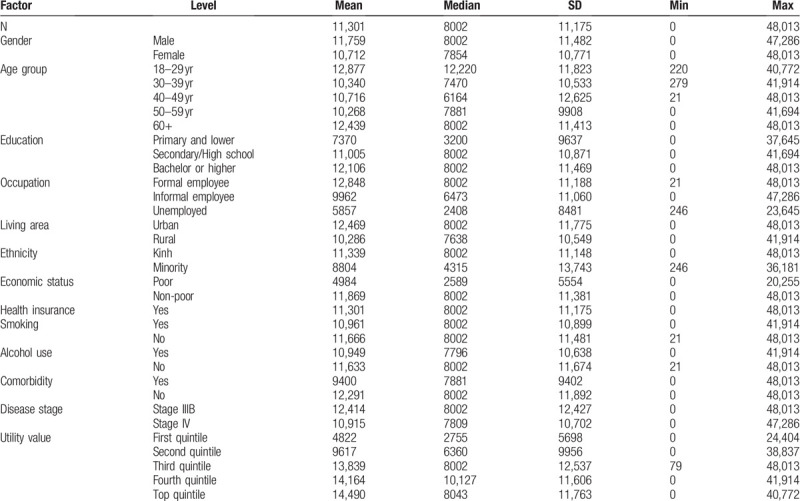
Willingness to pay for a quality-adjusted life year gained by patients’ characteristics.

The proportion of patients who were willing to pay for a QALY gained at the rate of equal or more than 1 GDP per capita of Viet Nam (USD $2342) was 79.0% (95% CI: 74.7–82.9%). This was higher among men, older patients, those with higher education, those working as formal employees, urban dwellers, Kinh people, non-poor people, non-smoking patients, non-drinking patients, patients without comorbidity, those at disease state IIIB and those with higher health utility (Table 4).

**Table 4:**
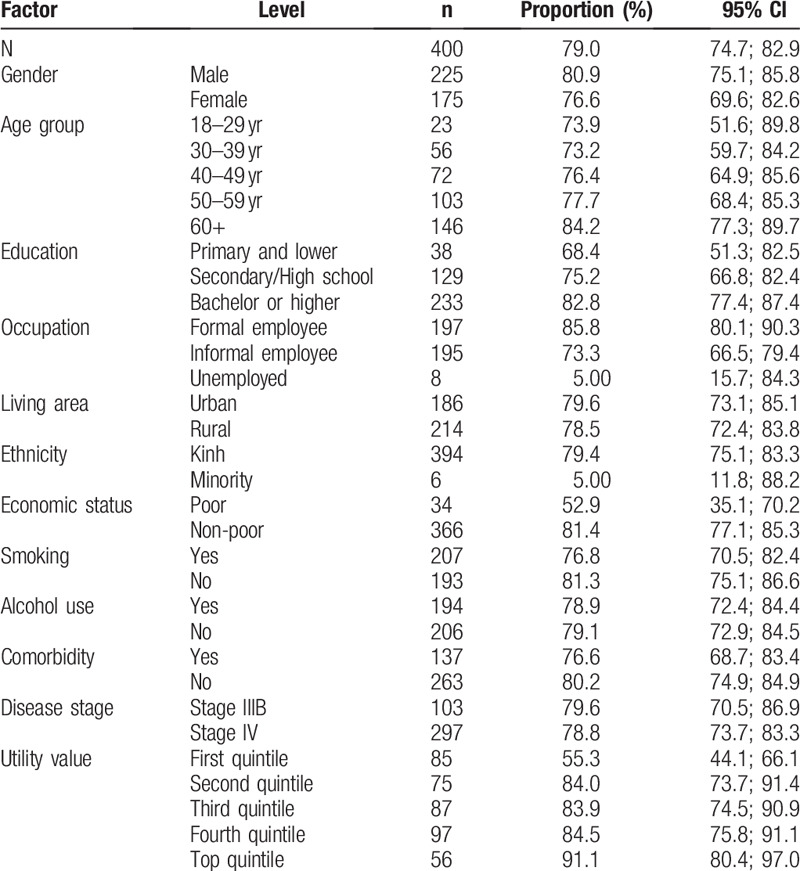
Patients having willingness to pay equal or above 1 gross domestic product by patients’ chacracteristics.

### 3.3 Regression analyses of correlates of the WTP/QALY

Gamma Generalized Linear Model (Table 5) shows that the WTP/QALY amount was significantly associated with respondent's

1) education – people with higher education were willing to pay a higher amount;
2) economic status – the non-poor people were willing to pay higher amount;
3) comorbidity status – people without the comorbidity were willing to pay higher amount; and
4) health utility – people with higher health utility were willing to pay higher amount.

**Table 5:**
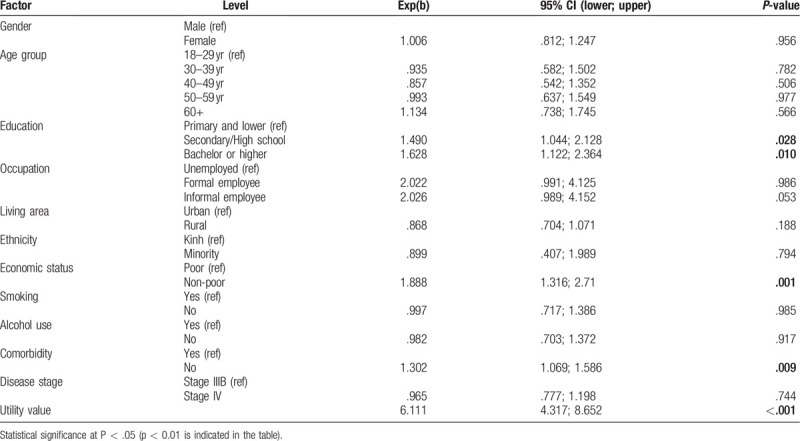
Gamma Generalized Linear Model for willingness to pay for a quality-adjusted life year gained.

Table 6 report identifies the multiple logistic regression analysis of correlates of willing to pay for a QALY gained at the rate of equal or more than 1 GDP per capita of Viet Nam. There was a strong correlation between willingness to pay for a QALY gained at the rate of equal or more than 1 GDP per capita of Viet Nam and economic status (the non-poor were willing to pay higher amount) and health utility (people with higher health utility were more likely willing to pay).

**Table 6:**
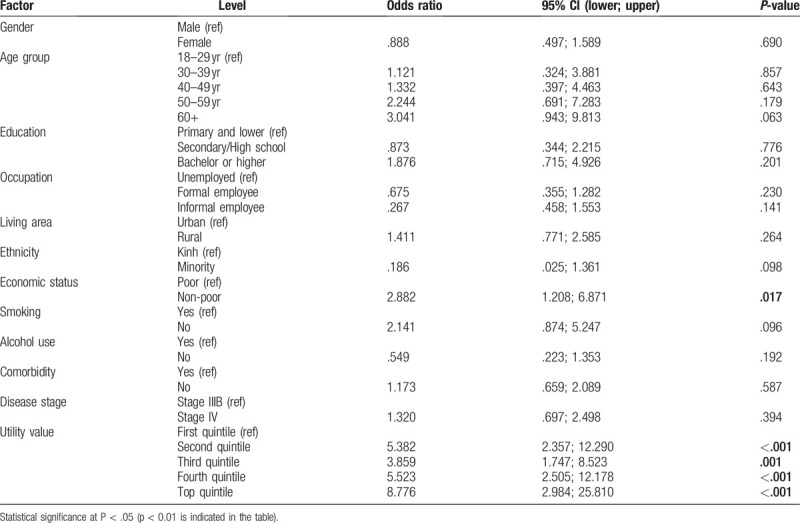
Multiple logistic regression for willingness to pay for a quality-adjusted life year gained at the rate of equal or more than 1 gross domestic product.

## 4 Discussion

To our knowledge, this is the first study in Viet Nam to analyze WTP for a QALY gained among advanced NSCLC patients. The evidence generated from this study may be useful for policymakers in prioritizing health interventions against NSCLC in Viet Nam.

Our study found that the overall mean WTP/QALY amount among NSCLC patients was USD $11,301. This is equal to about 4.4 GDP per capita of Viet Nam in 2017. This is much higher than the level of WTP/QALY among the general population in rural Viet Nam in 2012, which showed that the mean of WTP/QALY ranges from USD $667 to USD $993 (.38–.56 GDP per capita of Viet Nam in 2012).^[11]^ The WTP/QALY amount lies in the range of the treatment costs for lung cancers in Viet Nam in 2014 (VND 172,333,617–339,542,672 or USD $7833–15,434 for lung cancer stage III, and VND 160,690,121–266,197,825 or USD $7304–12,100 for lung cancer stage IV).^[3]^

The threshold of WTP/QALY among NSCLC patients in Viet Nam was higher than the thresholds reported from other Asian countries, with USD $8799 among patients with Epilepsy in China in 2010,^[12]^ USD $9000 among adults from the general population in Malaysia in 2014,^[13]^ and USD $5123 among patients with lung cancer in Thailand in 2015.^[14]^

The WTP/QALY amount found in this study was lower than the range of cost-effectiveness threshold of USD $25,971 to USD $38,964 (£20,000–30,000) used by National Institute for Health and Care Excellence (NICE) in 2008,^[15]^ and the most commonly cited threshold of USD $22,416 (€20,000) in the Netherlands.^[16]^ Higher results were derived from the existing values of preventing a statistical fatality in the UK context, with estimates ranging between USD $30,125 (£23,199) and USD $51,981 (£40,029) per QALY.^[17]^ In 2003, Gyrd-Hansen,^[18]^ using a discrete choice experiments approach and time-trade-off utilities, estimated a WTP per QALY of USD $13,448 (€12,000) in the general Danish population for relatively small-sized health gains. Shiroiwa et al^[19]^ study of WTP for an additional year of survival in full health found that the mean WTP per QALY ranged from USD $29,884 (£23,000) in the UK, USD $41,030 (€36,600) in Australia and USD $49,315 (€44,000) in the US.

Our findings suggest the significant association between WTP/QALY and the patient's education, economic status, comorbidity status. These findings are similar to the WTP/QALY among the general population in rural Viet Nam.^[11]^ A study from Thailand also showed that better-off people and those with a higher quality of life were significantly more likely to be interested in new treatment and be willing to participate in the treatment.^[14]^ The lower WTP was identified among worse-off patients who have a lower likelihood of accessing new treatment therapies. Thus, the Government of Viet Nam should provide further financial support to the disadvantaged groups in order to improve their access to life-saving treatments.

In this study, we found the health utility value is an independent factor of the WTP/QALY. A study conducted among metastatic breast cancer patients in Korea in 2009 also found the willingness-to-pay for cancer treatment was associated with higher quality of life score.^[20]^ However, this is different from the reports by some previous studies conducted among the general population in the UK in 1998,^[21]^ in Japan in 2011,^[22]^ and in Iran in 2015,^[23]^ which demonstrated that people with more severe health problems had higher value of WTP/QALY. The difference in the preference of the general population and that of the cancer patients could be an explanation for the difference in their willingness to pay. A study on the WTP/QALY among the general population in Vietnam should be conducted in the near future.

### 4.1 Methodological considerations

Some methodological constraint associated with the use of the contingent value method in this study was the potential bias introduced from the way the questions were framed, the contingent valuation scenarios, the elicitation method used, and the survey method that was conducted. To overcome these challenges, we conducted several field visits in order to develop appropriate contingent valuation scenarios and questions. We also implemented a number of cognitive interviews to make sure that the contingent valuation scenarios and questions were easy to understand among the local people. Appropriate training of enumerators and further field-testing also helped to ensure the validity and reliability of the study findings.

A disadvantage of the bidding model is the threat of starting-point bias, where the respondent's final WTP value is dependent on the first bid prompted by the interviewer.^[24,25]^ The starting-point bias is known as “an anchoring effect”^[26]^ which occurs when the first bid influences the WTP amount as the respondent may consider it as a “normal” value. We set up the starting point based on the experience of our pilot study.

The biggest limitation of this study is the convenience sampling. This is highly vulnerable to selection bias and high level of sampling error. Another limitation is information bias, which occurs when the WTP depends on who does the interview, what information is provided about the new treatment, and what other information the respondents have about the therapy. We selected interviewers with research experiences, and provided them with appropriate training to ensure they provide clear information about the treatment scenarios to minimize risk of bias.

The final limitation identified is strategic bias, which occurs when a respondent purposely states a higher WTP than the true level. We consider the risk of a strategic bias where respondents would overstate their true WTP as it is based on future predictions of treatment. A strategic bias where respondents would underestimate their true WTP would to the extent that it exists mean an underestimation of the elicited WTP in this study. Since the elicited WTP is high relative to the cost of provision, the risk of this bias does not present a substantial problem for this study.

## 5 Conclusions

In Viet Nam, lung cancer has a serious health and economic impact on patients, their families and the society. Estimating the WTP for a QALY gained threshold among NSCLC patients provides important information for the implementation of health technology assessment to prioritize health interventions in treating NSCLC in Viet Nam. Our study shows that many patients were willing to pay for the treatment that helps to improve their health. The amount of WTP/QALY ranged between the treatment cost, with WTP/QALY associated with socio-economic status and health status of the patient. Government and health policymakers should consider their ability to fund therapy for disadvantaged groups to ensure timely access to care.

## Acknowledgments

We thank physicians, administrative staff, and logistic staff at Bach Mai Hospital, Hanoi Oncology Hospital, Viet Nam National Cancer Hospital, Da Nang Hospital, Cho Ray Hospital, and Ho Chi Minh City Medicine and Pharmacy University Hospital for collaborating with us in the data collection process. We appreciate the language editing support from Ms. Nadera Rahmani from the Australian team at CENPHER.

## Author contributions

HVT, HVM, VQM, VNA, VVC, and DHL contributed to the study design, coordinating data collection in Viet Nam, developing research questions and conducting the statistical analysis of data, drafting and revising the manuscript; HTNA, KQL, PCP contributed to the data collection, conducting the statistical analysis of data, and drafting the manuscript; HTNA and KQL contributed to data analysis and drafting the manuscript. All authors read and approved the final submitted manuscript.

**Conceptualization:** Thuy Van Ha, Ngoc-Anh Thi Hoang, Mai Quynh Vu, Anh Nu Vu, Chinh Van Vu, Lieu Huy Duong, Minh Van Hoang.

**Data curation:** Anh Nu Vu, Chinh Van Vu, Lieu Huy Duong.

**Formal analysis:** Thuy Van Ha, Ngoc-Anh Thi Hoang, Mai Quynh Vu, Long Quynh Khuong, Minh Van Hoang.

**Funding acquisition:** Chinh Van Vu, Lieu Huy Duong.

**Investigation:** Anh Nu Vu, Pham Cam Phuong.

**Methodology:** Thuy Van Ha, Ngoc-Anh Thi Hoang, Mai Quynh Vu, Lieu Huy Duong, Minh Van Hoang.

**Project administration:** Anh Nu Vu, Pham Cam Phuong, Chinh Van Vu, Lieu Huy Duong, Minh Van Hoang.

**Resources:** Long Quynh Khuong, Anh Nu Vu, Pham Cam Phuong.

**Software:** Long Quynh Khuong, Pham Cam Phuong.

**Supervision:** Long Quynh Khuong, Pham Cam Phuong.

**Validation:** Long Quynh Khuong, Pham Cam Phuong.

**Visualization:** Long Quynh Khuong.

**Writing – original draft:** Thuy Van Ha, Ngoc-Anh Thi Hoang, Anh Nu Vu, Minh Van Hoang.

**Writing – review & editing:** Thuy Van Ha, Ngoc-Anh Thi Hoang, Mai Quynh Vu, Anh Nu Vu, Pham Cam Phuong, Chinh Van Vu, Lieu Huy Duong, Minh Van Hoang.

Minh Van Hoang: 0000-0002-4749-5536.
